# Acute and subacute macular and peripapillary angiographic changes in choroidal and retinal blood flow post-intravitreal injections

**DOI:** 10.1038/s41598-021-98850-8

**Published:** 2021-09-29

**Authors:** Nadhini Arumuganathan, Maximilian Robert Justus Wiest, Mario Damiano Toro, Timothy Hamann, Katrin Fasler, Sandrine Anne Zweifel

**Affiliations:** 1 Department of Ophthalmology, University Hospital Zurich, 8091 Zurich, Switzerland; 2 University of Zurich, 8006 Zurich, Switzerland; 3 Chair and Department of General and Pediatric Ophthalmology, Medical University of Lublin, 20079 Lublin, Poland

**Keywords:** Anatomy, Pathogenesis

## Abstract

Whether post injectional acute intraocular pressure (IOP) increase is associated with decreased peripapillary and macular perfusion is still under debate. Here, we investigated early changes in the choroidal and retinal blood flow using OCTA imaging in a cohort of patients undergoing anti-VEGF intravitreal injections (IVI) for macular edema following retinal vein occlusion and diabetic retinopathy. In this prospective single-center, observational study, the pre- and post-IVI changes in retinal perfusion were examined via assessment of vessel length density (VLD) and vessel density (VD) in deep and superficial capillary segmentations (DCP and SCP), foveal avascular zone (FAZ) in SCP, as well as flow signal deficits in the choriocapillaris segmentation. Mean IOP significantly changed over the study course (*p* = 0.000; ANOVA). Measurements at 5 min post-IVI (33.48 ± 10.84 mmHg) differed significantly from baseline (17.26 ± 2.41 mmHg, *p* = 0.000), while measurements from one day, one week, and one-month post-IVI did not (*p* = 0.907, *p* = 1.000 and *p* = 1.000 respectively). In comparison to baseline, no changes in OCTA parameters, including FAZ, VD, VLD, and FV, were detected 5 min post-IVI. No significant alterations in OCTA parameters were observed during study course. Increased IOP spikes were detected post-IVI; however, no potential permanent ischemic retinal damage was suspected.

## Introduction

To date, VEGF is considered to be the key factor in the process of angiogenesis, which is therapeutically targeted by anti-VEGF treatment modalities to significantly improve the visual function of affected patients^1,2^. Anti-VEGF injections (IVI) are used in several retinal disorders, including exudative age-related macular degeneration (nAMD)^3^, diabetic retinopathy (DR)^4^, retinal vein occlusions (RVO)^5,6^, and secondary choroidal neovascularization (CNV)^1,7^. Since 2006, the frequency of IVI administration has elevated to the point of becoming the second most common surgical procedure worldwide (after cataract surgery), with an estimated 5.9 million IVI cases in the USA alone in 2016^8,9^.

Anti-VEGF agents are known to be efficient as well as safe. However, anti-VEGF agents are short-acting drugs, and a single anti-VEGF treatment is generally not sufficient for elimination of pathological lesions. Given the need for frequent injections, particularly in DR and nAMD cases, novel reports of long-term risks have now been surfacing^10^.Previous studies have shown that immediately after IVI, intraocular pressure (IOP) increases^11,12^. The increased IOP values have been observed to normalize within one day^13–15^. On the other hand, long-term studies have shown that, besides this short-term IOP increase, IVI may lead even to long-lasting IOP elevations^16^ and repeated episodes of IOP spikes might lead to glaucoma progression^17,18^. Moreover, short- and long-term studies shown that high IVI frequency might also be associated with a higher risk of elevated IOP^10,19–21^. Indeed, considering that multiple IVIs are often required due to the chronic course of the retinal diseases, a cumulative long-term effect of IOP elevation may affect the macula and the optic nerve^22,23^. As a consequence, hypotensive treatments, or even surgical glaucoma therapy, might become necessary^24^.

Optical coherence tomography angiography (OCTA) is a non-invasive infrared imaging technique^25^, which is used for angiographic and structural analyses, such as intravenous dye injection^26^. OCTA is used for quantitative assessment of microvasculature, such as vessel length density (VLD) and vessel density (VD)^27–29^. In addition, OCTA allows clear visualization of choroidal and retinal microvasculature, and can be used for diagnostic, therapeutic, as well as monitoring purposes^30–32^.

Recently, Barash et al. detected an acute increase in IOP and a reduction of peripapillary and macular perfusion density using OCTA immediately post-IVI in a heterogenous group of patients^33^. However, the question of whether the post-IVI acute IOP increase is associated with decreased peripapillary and macular perfusion is still controversial. Therefore, increased IOP due to IVI remains debatable, despite its generally favorable safety profile^9^.

In this study, we aimed to qualitatively and quantitatively investigate early changes in retinal and choroidal blood flow using OCTA imaging in a homogeneous cohort of patients undergoing anti-VEGF IVI for macular edema following DR and RVO.

## Methods

This prospective single-center, observational study was performed at the Medical Retina Clinic, University Hospital of Zurich, Zurich, Switzerland. The study cohort consisted of all patients diagnosed with macular edema following DR or RVO and undergoing anti-VEGF IVI treatment along with aflibercept or ranibizumab between August 2018 and March 2019. The Institutional review board approved the study protocol (Cantonal Ethics Committee, Canton of Zurich, BASEC nr.2018-00961). We followed the guidelines of the Declaration of Helsinki. All patients provided a written informed consent.

### Inclusion and exclusion criteria

Inclusion criteria were: a macular edema secondary to DR or RVO treated with anti-VEGF-therapy (Aflibercept or Ranibizumab), an age ≥ 18 years old and a best corrected visual acuity (BCVA) > 20/100. Exclusion criteria were: all ocular comorbidities precluding the proper acquisition of OCTA images (signal strength of 7/10 or lower), such as corneal or media opacities, previous ocular trauma or surgery or presence of glaucoma; physical limitations that prevent from sitting on a chair for about 30 min, keeping the head and eyes in a relaxed position (for example, general immobilization, stiffened cervical spine, nystagmus, etc.); a high myopia (> 4 diopters).

### Clinical assessment

All patients underwent an ophthalmological examination with best-corrected visual acuity (BCVA), IOP measurements, slit lamp examination and fundus biomicroscopy, swept-source OCTA, and spectral-domain (SD) OCT. Additionally, vital signs, such as systolic and diastolic blood pressures and pulse rate were also acquired. The HbA1C value was acquired from electronic health records (eHR).

All examinations were performed at baseline, and 1 day, 1 week, and 1-month post-IVI treatment. IOP, OCTA, and vital parameters were also acquired immediately post-IVI.

Patient data, such as age, gender, general medical history, diagnosis, ophthalmological findings, as well as the previous number of injections were acquired from electronic health records (eHR) and subjected to statistical analysis. Early Treatment Diabetic Retinopathy Study (ETDRS) charts were sued to assess BCVA^34^. Immediately after each IVI, the uncorrected visual acuity (UCVA) was performed to assess the hand movement.

IOP (mmHg) measurements were acquired using Tonopen (Tono-Pen AVIA®, Reichert, New-York, USA). An IOP spike was defined as an increase in IOP of more than 20% from the baseline.

OCT images were examined via an SD Heidelberg Spectralis device v. 1.9.10.0 (Heidelberg Eye Explorer 2, Heidelberg Engineering, Heidelberg, Germany). A 20 × 35° macular cube scans with 25 averaged frames and 32 B-scans were obtained. In addition, OCT-EDI was also captured.

### OCT imaging

OCTA images were obtained using the swept-source PLEX Elite 9000 device, software version 2.0.1.47652 (Carl Zeiss Meditec Inc. Dublin, CA, USA). To detect the effect of IVI on retinal structures and blood flow, 3 mm × 3 mm and 6 mm × 6 mm cube scans centered on the macula and 6 mm × 6 mm cube scans centered on the optic nerve were acquired. En face OCTA images of the retinal vasculature were generated from the SCP and DCP after automatic projection artifact removal (PAR) which removes the projection artifacts of the SCP in the images of the DCP using the software supplied by the manufacturer (ARI Network Hub, Carl Zeiss Meditec Inc., Dublin, CA, USA)^29^.

Scans with signal strength of ≤ 8 of 10 were included for further analysis^35^. Furthermore, scans with incorrect centration, out-of-focus artifacts, motion artifacts or failed PAR were excluded from further analysis. The analysis of the vessel density (VD), vessel length density (VLD) in the deep and superficial capillary segmentations (DCP and SCP), and foveal a vascular zone (FAZ) in the SCP were performed with Image J (National Institutes of Health, Bethesda, Maryland, USA) using a threshold algorithm for binarization and similarly to previous studies, according to the methods previously described^36,37^. Additionally, flow signal deficits (FD) in the choriocapillaris (CC) segmentation were analyzed. CC flow signal deficits (FD) were assessed as the fraction of the area without any flow signal in binarized CC segmentation slabs. The CC analysis was performed using en face OCTA slab with a thickness of 20 µm and offsets 9 μm and 29 μm below the automated retinal pigment epithelium (RPE) segmentation. Binarization was performed using Image J (National Institutes of Health, Bethesda, Maryland, USA), using a threshold algorithm as previously described^36,37^. The analyzed areas of interest were a circle with a diameter of 0.5 mm (area1), centered on the fovea and 1 concentric ring (area2) with an inner radius of 0.5 mm and an outer radius of 1 mm, as already reported in previous literature^38^. Area 1 comprised of a scanned circle of CC slab, centered on the fovea, with a diameter of 0.5 mm and an area of 0.196 mm^2^, which was meant to analyze the CC FD within a standardized FAZ. VD is the percentage of the image area covered by the flow signal. VLD is defined as the total length of vessels per square mm (in mm^-1^). FD represents the area of the CC segmentation that is not covered by the flow signal.

### Statistical analysis

To visualize data, pre- and post-IVI measurements of IOP and OCTA parameters were plotted for each patient individually and displayed using lattice plots. Using a paired t-test, OCTA and IOP values pre- and immediately post-IVI were compared. In addition, analysis of covariance (ANCOVA)^39^ was performed to assess the influence of IOP on OCTA parameters 5 min post-IVI as the outcome. BCVA values at baseline and follow-up visits were compared using ANOVA; if the differences were significant, the values were analyzed using Tukey HSD (Honest Significant Differences) test. All reported data is displayed as mean value ± standard deviation (SD). Statistical significance was indicated by a *p*-value < 0.05.

Color-coded heat maps were used for the qualitative assessment of vascular drop-outs. The retinal perfusion density (calculated using VD) and vessel density (calculated using VLD) were generated for SCP and DCP. A color-scale was used, where blue represents low VD or VLD and red represents high VD or VLD.

## Results

In this study, 33 patients were recruited. Overall, four (12.12%) patients were excluded from the final analysis, of which two patients were excluded due to insufficient image quality and the other two patients were excluded due to segmentation failures post-processing. Twelve (41.38%) patients with DR and 17 (58.62%) patients with RVO met the inclusion criteria. The baseline data are summarized in Table 1.

**Table 1:** Patient demographics of study cohort at baseline.

	DR	RVO	*p*-value*
**Gender**			
Male	7	11	
Female	5	6	
Mean age (years) (± SD)	58.62 (± 15.07)	70.19 (± 10.91)	0.024
Mean BCVA (± SD)	77.54 (± 9.18)	74.31 (± 13.73)	0.475
Mean IOP (± SD)	17.15 (± 2.58)	17.56(± 2.68)	0.681
Mean n° of IVI (± SD)	18.08 (± 19.65)	24.56 (± 16.15)	0.338

*DR* diabetic retinopathy, *RVO* retinal vein occlusion, *SD* standard deviation, *BCVA* best corrected visual acuity, *n°* number, *IOP* intraocular pressure, *IVI* intravitreal injection. *t-test comparing means of both subgroups.

At baseline, mean BCVA (± SD) was 75.26(± 12.09). No significant differences in BCVA over the study course, compared to the baseline, were detected during the ANOVA analysis (*p* = 0.988, *p* = 0.957 and *p* = 0.996 at 1 day, 1 week and 1 month respectively).

Mean IOP changed significantly during the study period (*p* = 0.000; ANOVA). Tukey HSD showed that values at 5 min post-IVI (33.48 ± 10.84 mmHg) differed significantly from baseline (17.26 ± 2.41 mmHg, p = 0.000), while those from one day, one week, and one-month post-IVI did not (*p* = 0.907, *p* = 1.000 and *p* = 1.000 respectively). These results are summarized in Table 2.

**Table 2:** Comparison of mean IOP and mean BCVA.

	Baseline	5 min	1 day	1 week	1 month	ANOVA *p*-value
Mean IOP (mmHg) ± SD	17.26 ± 2.41	33.48 ± 10.84*	15.96 ± 2.90	17.37 ± 2.80	17.19 ± 3.19	0.000
Mean BCVA (ETDRS letters) ± SD	75.26 ± 12.09	–	74.29 ± 10.46	76.74 ± 10.22	75.93 ± 10.09	0.861

*IVI* intravitreal injections, *ANOVA* analysis of variance, *IOP* intraocular pressure, *mmHg* millimeters of mercury, *BCVA* best corrected visual acuity, *ETDRS* early treatment diabetic retinopathy study, *SD* standard deviation.

*Tukey Honestly Significant Difference (HSD) versus baseline (*p* < 0.001).

**Figure 1:**
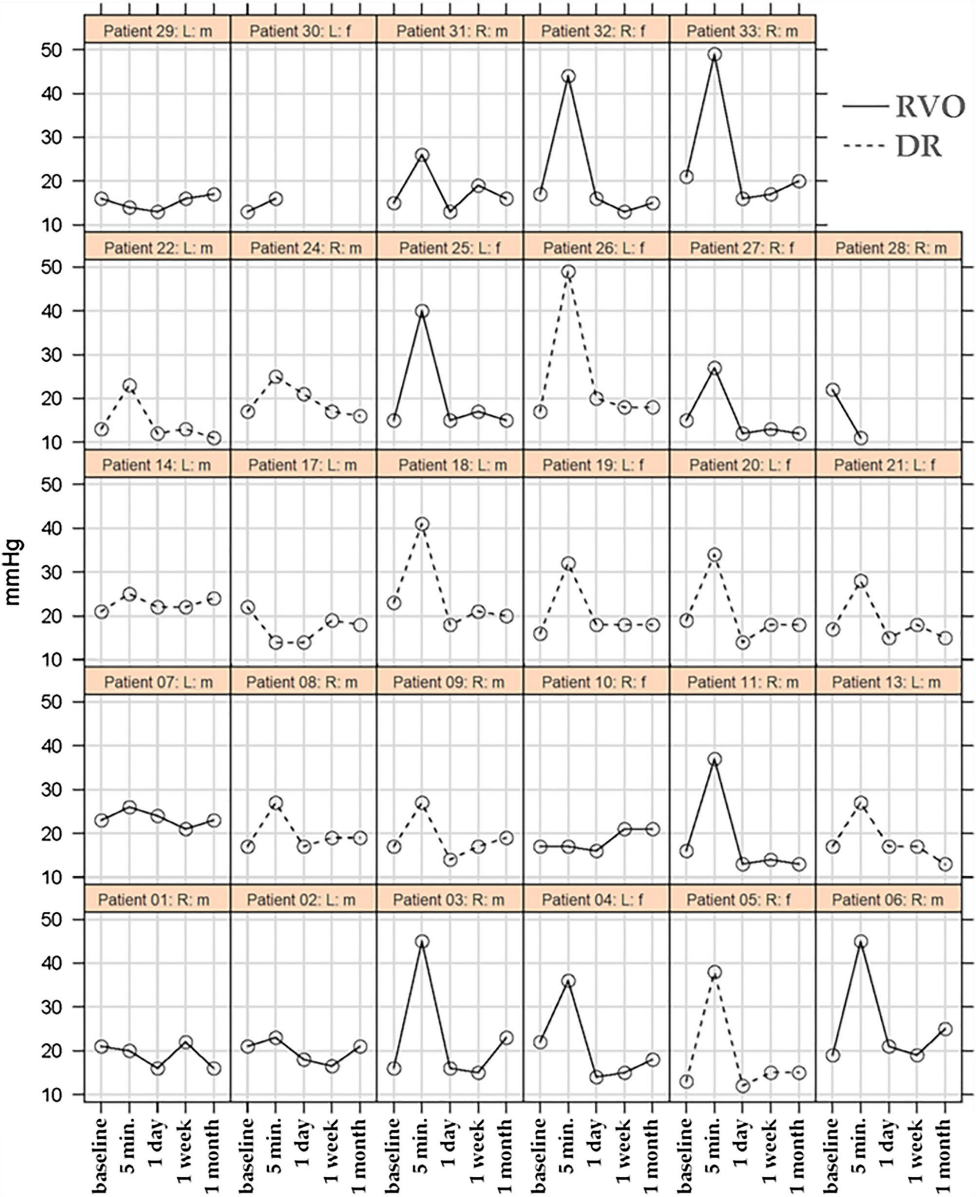
Lattice plot of intraocular pressure (IOP) at baseline and 5 min, 1 day, 1 week, and 1-month post-injection. RVO: retinal vein occlusion; DR: diabetic retinopathy; mmHg: millimeters of mercury; min: minutes; R: right eye; L: left eye; m: male; f: female.

Figure 1 shows the individual IOP measurements of each patient (baseline and 5 min, 1 day, 1 week, and 1-month post-IVI).

No significant changes in quantitative OCTA parameters, including FAZ, VD, VLD, and FV, were observed at 5 min post-IVI. Mean change in scores are depicted in Table S1 (Supplementary material).

The ANCOVA analysis showed that none of the assessed variables (VD, VLD, FAZ, and FV) were significantly associated with IOP. ANCOVA coefficients and confidence intervals are displayed in Table S1 (Supplementary material).

### Qualitative heatmap analysis

**Figure 2:**
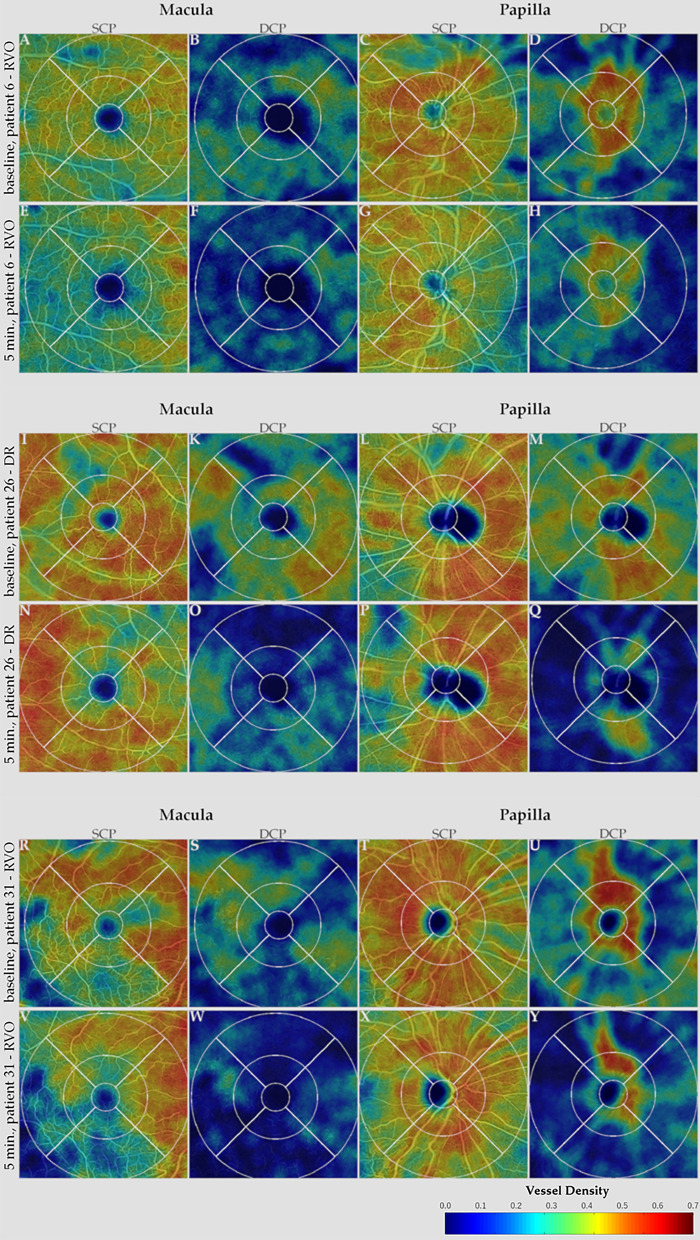
Sequential optical coherence tomography angiography (OCTA) heatmaps representing vessel density (VD) of patient #6 (RVO), #26 (DR), and #31 (RVO). Patient #6 (**A**–**H**), patient #26 (**I**–**Q**), and patient #31 (**R**–**Y**) show superficial and deep capillary plexus (SCP, DCP) segmentation of macular and papillary scans at baseline and 5 min post-injection. Panels (**A**–**D**, **I**–**M**, and **R**–**U)** show VD in SCP and DCP at baseline in patient#6, #26, and #31, respectively. At 5 min post-injection (**E**–**H**, **N**–**Q**, **V**–**Y**), when intraocular pressure (IOP) was 45, 49, and 26 mmHg in patient #6, #26, and #31, respectively, a diffuse reduction in VD can be observed in all quadrants of the macular (**E**–**F**, **N**–**O**, **V**–**W**) and papillary (**G**–**H**, **P**–**Q**, **X**–**Y**) panels. For all patients, only a slight focal reduction can be seen in the SCP (**E**, **G**, **N**, **P**, **V**, **X**), while the reduction in VD is more severeas depicted in DCP heatmaps (**F**, **H**, **O**, **Q**, **W**, **Y**).

After IVI, 24 out of 29 patients (82.76%) exhibited an IOP increase and 20 (68.97%) exhibited an IOP spike. Seventeen patients (70.83%) who exhibited an increase in IOP presented a qualitative decrease in the perfusion-based heatmap. Representative perfusion map samples showing a high variability of perfusion patterns in our study cohort, which reflects different disease stages and IOP change patterns, are shown below in Fig. 2.

## Discussion

In this study cohort, we analyzed the qualitative and quantitative changes in OCTA parameters in patients with DR and RVO post-IVI. No changes in quantitative OCTA parameters, including FAZ, VD, VLD, and FV were observed 5 min post-injection compared to baseline and during the study course. During the qualitative analysis, 16 of 29 (55.17%) patients showed decreases in VD in the heat maps (Fig. 2).

IOP spikes are likely to occur in certain glaucoma cases as well as post-IVI 39^40,41^. However, the impact of these transient IOP elevations on retina physiology is poorly understood^41^.

Some authors have shown that high IOP spikes and changes in OCTA parameters can be observed post-IVI^9,33^.Recently, Barash et al. detected an acute increase in IOP and a reduction of peripapillary and macular vessel density reflected via changes in OCTA parameters immediately post-IVI in a heterogenous group of patients^33^.Additionally, animal studies have reported that the restriction in blood supply often results in vessel occlusions and irreversible retinal damage^42,43^. In a study on monkeys, Cheung et al. showed that IOP spikes affected the whole retinal and choroidal blood flow and focal defects in perfusion persist beyond the actual IOP spikes. Also in our cohort, seventeen patients (70.83%) who exhibited an increase in IOP presented a qualitative decrease in the perfusion-based heatmaps within 5 min after the injection (Fig. 2). However, contrary to the results shown by Cheung et al. that observed a marked reduction in flow signal in the DCP and in the CC layers, for all our patients, only a slight focal reduction can be seen in the SCP, while the reduction in VD is more severe as depicted in DCP heatmaps. These persistent defects might result in an ischemic insult of chorioretinal tissue and lead to long-term damage to the retina and the choroid. Pressure-related ocular damage might be attributed to direct or indirect effects on tissue perfusion owing to the elevated IOP^9^. Indeed, in case of reduced perfusion, it is hypothesized that based on the concept of the serial model of retinal capillary plexus, the primarily venous and efferent location of the DCP may reside in an environment of the lowest oxygen tension and perfusion pressure, making it most susceptible to ischemic injury. Conversely, the primarily arterial, afferent SCP would be most resistant to ischemic injury and last to be affected^44,45^.

Another animal study reported long-term ganglion cell dysfunction, as well as evidence of vascular remodeling in the SCP and intermediate vascular plexus in mice post-IOP spikes^41^. Interestingly, the mouse model showed no difference in DCP, in contrast to the monkey model of Cheung et al., where the DCP was the most affected vascular plexus^9,41^. In our cohort, we also observed a significant increase in IOP from 17.26 ± 2.41 mmHg at baseline to 33.48 ± 10.84 mmHg at 5 min post-injection (*p* = 0.000) and an IOP spike in 20 of 29 patients. We were unable to observe significant any influence of IOP change on our OCTA parameters in ANCOVA. However, when reviewing VD heat maps, decreases in VD, especially in the DCP, could be observed, even if the quantitative analysis did not show any significant changes in the overall cohort. This might be due to lower IOP spikes in our study compared to other human^33^ or animal studies^9^. Indeed, our IOP spikes ranged from 26 to 49 mmHg, while the previously studies reported a mean IOP post-injection of 46.35 ± 12.15 mmHg with a maximum of 72 mmHg^33^ and IOP spikes of over 90 mmHg^9^.

Previous studies have shown that increased IOP values normalize within one day^13–15^. In our study, IOP spikes resolved in all 20 patients after 1 day. El Chehab et al., in a prospective study on 30 IVIs, demonstrated that 40% of patients exhibited an IOP of greater than 45 mmHg 1 min post-injection and a mean IOP value of 23.6 ± 2.1 mmHg at 5 min post-IVI^13^. In our series, we did not check IOP at 1 min post-IVI, while at 5 min post-IVI, mean IOP was 33.48 ± 10.84 mmHg with an IOP spike in 68.97% of patients.

Evidence has suggested a possible vascular component to play a key role in the pathogenesis and progression of glaucoma^46^. This vascular theory postulates that decreased blood flow to the retina leads to retinal ganglion cell death^46,47^. Rao et al. have shown that VD reduction on OCTA reaches a base level at a more advanced disease stage and, therefore, can be used to monitor advanced glaucomatous damage in eyes^47^. Lee et al. have shown that the instant IOP elevation post-IVI leads to a transient decrease in mean ocular perfusion pressure but did not impair retinal blood flow significantly^11^. A previous meta-analysis has also shown a decrease in retinal nerve fiber layer (RNFL) thickness 12 months post-IVI, but could not determine its clinical relevance^23^. In our study, we did not detect any changes in OCTA parameters, especially in the FAZ, VD, VLD, and CC FD. However, we did not investigate RNFL dynamics in our cohort.

Certain risk factors for the occurrence of IOP spikes have been identified in the previous literature. Gismondi et al. have shown that IVI causes a transient IOP rise. The IOP increase can be detected in both phakic and pseudophakic eyes, with hyperopic eyes more affected^16^. Pallikaris et al. and Kotliar et al. further identified the injected volume, scleral thickness, scleral rigidity, and globe size as being related to the IOP increase^48,49^. Cheung et al. have postulated that the volume of injection of anti-VEGF agents may lead to ischemia due to elevated IOP, but the pharmacological effect of these agents may prevent some of the potential ischemic injury due to higher IOP and post-ischemic VEGF-secretion. It remains to be seen if there is cell damage related to recurrent vascular defects, if the vascular defects may fully recover, or whether the retinal vasculature may become increasingly susceptible to irreversible damage with repeated episodes of IOP rise in patients who received multiple IVI, especially in at-risk groups such as those with glaucoma or pre-existing vasculopathy. Sustained release of anti-VEGF agents may obviate these possibilities^9^. According to Loureiro et al., IVI caused a significant rise in IOP, irrespective of the needle size used. The 27-gauge needle coursed with lower post-injection IOP without prejudice of the patient comfort^12^. They hypothesized that the lower post-injection IOP values with 27-gauge needle might be attributed to a higher rate as opposed to smaller needles, as reported by Pang et al.^50^. Hence, Pallikaris et al. hypothesized that larger 27-gauge needles are less associated with IOP spikes, as they might cause a higher vitreous reflux^48^. In our series, even if we used a 30-guage needle in a standardized procedure and a straight scleral injection technique performed by two retinal specialists (S.Z., K.F.), a vitreous reflux could not be excluded^51^ and could also justify the lower post-injection IOP values, compared to the results of Barash et al., although they do not specify their injection technique^33^.Indeed, previous authors have shown that the injection technique is linked with IOP increase post-IVI and IOP was higher when a tunneled scleral injection was performed with a mean IOP of 31.8 ± 14.0 mmHg at 3 min post-IVI in the straight injection group, which corroborated our results^52^.

The main limitations of this study included the small sample size, the heterogeneous study population with regard to age, the diagnosis with inclusion of only the patients affected by DR and RVO, the absence of data about the cumulative effect of repeated IVI on OCTA parameters, the absence of treatment naïve patients and of measurements 1 min post-IVI.

In conclusion, we were unable to detect any changes in OCTA parameters during our study course. We verified higher IOP spikes post-IVI and could not rule out any potential permanent ischemic retinal damage based on our findings. However, it is not possible to exclude a cumulative effect of IVI on OCTA parameters and IOP on a long-term follow-up. Multiple IVI could lead to persistent ocular hypertension. Physicians should be aware of this condition and monitor their patients for persistent ocular hypertension, especially in eyes with preexisting glaucoma and repeated IVI. Rapid diagnosis and treatment can help reduce the risk of vision loss^53^. Thus, further randomized controlled studies with a longer follow-up and a larger, more heterogeneous sample size are highly warranted.

## Supplementary Information

Supplementary Information.
