# Supplementary material for: Acute and subacute macular and peripapillary angiographic changes in choroidal and retinal blood flow post-intravitreal injections
Source: Sci Rep. 2021 Sep 29;11:19381. doi: 10.1038/s41598-021-98850-8 (PMC8481476; doi:10.1038/s41598-021-98850-8)
Supplement: Supplementary file 1 — Supplementary Information. [file 41598_2021_98850_MOESM1_ESM.docx]

# Acute and subacute macular and peripapillary angiographic changes in choroidal and retinal bloodflow post-intravitreal injections

Nadhini Arumugunathan^1^, Maximilian Robert Justus Wiest ^1^, Mario Damiano Toro ^1,2^, Timothy Hamann^1^, Katrin Fasler^1^ and Sandrine Anne Zweifel^1,^*

*Corresponding Author

| **OCTA parameter** | **Mean change score** | **SD** | **Coefficient IOP at 5 min.** | **CI** | **Coefficient diff. IOP** | **CI** |
| --- | --- | --- | --- | --- | --- | --- |
| *VD.S3* | -0.011 | 0.02 | -0.00014 | -0.0026 to 0.0029 | -0.00113 | -0.0037 to 0.0014 |
| *VD.D3* | -0.007 | 0.02 | -0.00107 | -0.0015 to 0.0036 | -0.00126 | -0.0037 to 0.0011 |
| *VLD.S3* | -0.533 | 1.22 | -0.02958 | -0.1078 to 0.1670 | -0.08236 | -0.2081 to 0.0434 |
| *VLD.D3* | -0.287 | 0.83 | -0.07387 | -0.0286 to 0.1764 | -0.08154 | -0.1779 to 0.0148 |
| *VD.S6* | -0.004 | 0.02 | -0.00121 | -0.0019 to 0.0043 | -0.00101 | -0.0040 to 0.0019 |
| *VD.D6* | -0.002 | 0.02 | -0.00155 | -0.0013 to 0.0044 | -0.00139 | -0.0041 to 0.0013 |
| *VLD.S6* | -0.090 | 0.91 | -0.01964 | -0.1430 to 0.1037 | -0.01288 | -0.1048 to 0.1306 |
| *VLD.D6* | -0.102 | 0.93 | -0.04602 | -0.0842 to 0.1763 | -0.04317 | -0.1670 to 0.0807 |
| *VD.S6.pap* | -0.006 | 0.03 | -0.00003 | -0.0041 to 0.0042 | -0.00068 | -0.0033 to 0.0046 |
| *VD.D6.pap* | -0.003 | 0.04 | -0.00019 | -0.0064 to 0.0061 | -0.00009 | -0.0058 to 0.0060 |
| *VLD.S6.pap* | -0.240 | 1.12 | -0.05898 | -0.2274 to 0.1094 | -0.07546 | -0.0859 to 0.2368 |
| *VLD.D6.pap* | -0.106 | 1.24 | -0.01258 | -0.1572 to 0.1823 | -0.01452 | -0.1766 to 0.1476 |
| *FV.pap.ir* | -0.597 | 2.72 | -0.20310 | -0.3183 to 0.7245 | -0.28420 | -0.7679 to 0.1994 |
| *FV.pap.or* | -1.065 | 3.43 | -0.00851 | -0.7037 to 0.6867 | -0.09498 | -0.7364 to 0.5465 |
| *FAZ* | -0.005 | 0.02 | -0.00024 | -0.0026 to 0.0031 | -0.00099 | -0.0037 to 0.0018 |

**Supplementary material**

**Table S1.** Overview of mean change scores, coefficients with IOP at 5 minutes and coefficients with change in IOP in between baseline and 5 minutes after injection.

Abbreviations: OCTA: optical coherence tomography angiography; SD: standard deviation; IOP: intraocular pressure; 5 min.: 5 minutes after injection; CI: confidence interval; diff. IOP: differential in IOP in between baseline and 5 min.; VD: vesseldensity; VLD: vessel length density;S3: Superficial capillary plexus in 3mm x 3mm macular image; S6: Superficial capillary plexus in 6mm x 6mm macular image; S6.pap: Superficial capillary plexus in 6mm x 6mm peripapillary image; D3: Deep capillary plexus in 3mm x 3mm macular image; D6: Deep capillary plexus in 6mm x 6mm macular image; D6.pap: Deep capillary plexus in 6mmx6mm peripapillary image; FV: flow voids; pap.ir: inner peripapillary ring; pap.or: outer peripapillary ring; FAZ: Foveal avascular zone.
